# Special Issue “Latest Research in Post-COVID (Long COVID): Pathological and Treatment Studies of Sequelae and Complications”

**DOI:** 10.3390/biomedicines12061188

**Published:** 2024-05-27

**Authors:** César Fernández-de-las-Peñas

**Affiliations:** Department of Physical Therapy, Occupational Therapy, Physical Medicine and Rehabilitation, Universidad Rey Juan Carlos (URJC), 28922 Alcorcón, Madrid, Spain; cesar.fernandez@urjc.es; Tel.: +34-91-488-88-84

The Severe Acute Respiratory Syndrome Coronavirus 2 (SARS-CoV-2) pathogen provoked the most unprecedented sanitary outbreak of the current century by causing coronavirus disease 2019 (COVID-19), which has led to approximately 775 million confirmed cases and more than 7 million deaths globally [1]. The COVID-19 outbreak has also prompted one of the most significant explosions of research in the last century, as thousands of papers have been published in a short period of time (four years). In fact, the extensive literature concerning COVID-19 has concentrated on the management of acute cases [2] and the prevention of the spread of the virus, e.g., vaccines [3].

Despite every endeavor to fight against COVID-19, the world is now confronted by another escalating healthcare problem derived from the outbreak: the development of long-lasting symptoms once the acute SARS-CoV-2 infection has passed. The presence of long-lasting symptoms after acute infection has been named long-COVID [4] or post-COVID-19 condition [5]. In fact, more than 100 post-COVID-19 symptoms have been attributed to SARS-CoV-2 infection [6]. Current data suggest that up to 25–30% of COVID-19 survivors report long-lasting post-COVID symptoms at one [7,8] and two [9,10] years after the infection. However, several aspects of this condition remain unknown, such as its underlying mechanisms, its consequences, and treatment strategies for the management of these patients.

This Special Issue of *Biomedicines*, entitled “Latest Research in Post-COVID (Long-COVID): Pathological and Treatment Studies of Sequelae and Complications”, focused on these aspects of post-COVID-19 condition, a topic of emerging relevance due to the expected presence of millions of “long-haulers”. A total of fourteen papers were published in this Special Issue, with the following topics addressed: (1) the treatment of post-COVID-19 condition; (2) the repercussions of SARS-CoV-2 infection in neonates; (3) risk factors of severe COVID-19; and (4) the phenotyping of post-COVID pain.

**Treatment** **of Post-COVID-19 Condition**

The development of treatment strategies for post-COVID-19 condition is an important topic. Among the plethora of post-COVID symptoms that a COVID-19 survivor may suffer from, fatigue and dyspnea are likely the most bothersome. Various studies have reported a prevalence of post-COVID fatigue ranging from 32% [11] to 42% [12] in the first six months after infection, and a prevalence of post-COVID dyspnea ranging from 26% to 41% [13].

The meta-analysis conducted by Meléndez-Oliva et al. reported the moderate to large effects that pulmonary rehabilitation has on post-COVID dyspnea, but not on fatigue, physical function, quality of life, and depressive symptoms; this was in comparison to the typical care interventions (n = 34 trials) [14]. Most studies included in this meta-analysis used exercise and breathing retraining as the main components of pulmonary rehabilitation [14]. Therefore, a potential explanation for this lack of effect on post-COVID fatigue could be that the exercise administered was not personalized to each patient and, accordingly, that the intensity or volume of exercise was not sufficient to reach fatigue levels; in addition, breathing retraining exercises are mainly focused on the respiratory, but not cardiovascular, system. In fact, the systematic review presented by Sánchez-García et al. found limited evidence that physical therapy interventions related to respiratory musculature and moderate-intensity exercise led to significant improvements in post-COVID fatigue and dyspnea [15]. In fact, it seems that intense exercise, e.g., high-intensity interval training (HIT), moderate-intensity continuous training and strength training, can effectively enhance skeletal muscle deconditioning in patients with post-COVID-19 condition [16]; however, such exercise programs should be individualized due to the presence of post-exertional malaise in this population [17].

In addition to rehabilitation, the treatment of post-COVID-19 condition involves the utilization of other interventions. Among these potential interventions, a study published in this Special Issue found that the administration of micronized Palmitoylethanolamide plus Luteolin (CoUltraPEALut) as an adjuvant treatment with olfactory training effectively aids in the overall recovery of post-COVID olfactory problems (anosmia, hyposmia) [18]. Other medications, such as nonsteroidal anti-inflammatory drugs (NSAID), have been proposed for the management of some symptoms of post-COVID-19 condition. The rationale for applying NSAIDs is based on a reduction in the proinflammatory cytokine response associated with the infection; however, the early application of NSAIDs, at least in the acute COVID-19 phase, has been hypothesized to negatively impact the initial antiviral immune response of the host [19]; however, this hypothesis needs to be further investigated. In this Special Issue, Gyöngyösi et al. describe an improvement in the clinical symptomatology associated with a decrease in the presence of cardiac abnormalities (probably due to ongoing myocardial inflammation) with the prolonged use of NSAIDs in individuals with post-COVID cardiac symptoms [20].

**Neonatal** **Repercussion of SARS-CoV-2 Infection**

The risk of the potential perinatal transmission of SARS-CoV-2 has received particular attention from the beginning of the outbreak. With the rapid development of COVID-19 vaccines, questions concerning the safety of vaccination during pregnancy have been raised. Overall, vaccination during pregnancy does not seem to be associated with an increased risk of adverse pregnancy or perinatal outcomes [21].

However, the risk of the transmission of SARS-CoV-2 infection during pregnancy remains unclear. In fact, at the beginning of the pandemic, it was documented that SARS-CoV-2 can result in a high incidence of premature birth, miscarriages or maternal death [22]. This information has changed with further research. Thus, a systematic review published in this Special Issue investigates the possibility of vertical transmission from mother to child [23]. This study found that vertical transmission from mother to child during pregnancy (i.e., transmission via placenta) is not supported by current data, but that vertical transmission at the time of delivery or breastfeeding can be exceptionally possible [23]. Other reviews have also been unable to identify any significant association between acute SARS-CoV-2 infection in early pregnancy (the first 20 weeks of gestation) and adverse fetal, neonatal or maternal outcomes [24,25]. Nevertheless, Rodriguez-Wallberg et al. warned of a 44% increase in the rate of miscarriage rate in recent years [24]. Similar results were also identified by Brandibur et al., who reported that SARS-CoV-2 acute infection during pregnancy was unlikely to cause congenital digestive malformations; however, these authors observed that the number of gastrointestinal malformations was higher during 2022 (n = 47) than during the 3 years (2017–2020) prior to the COVID-19 outbreak (n = 39) [26].

**Risk** **Factors of Severe COVID-19**

The identification of individuals at a higher risk of developing severe COVID-19 has received particular attention in the literature. In fact, several studies have investigated whether the presence of deficiencies in gene expression could lead to a higher risk of experiencing the severe form of this condition. For instance, Saengsiwaritt et al. revealed that subjects carrying the C allele of the transmembrane protease serine-2 (TMPRSS2) rs12329760 polymorphism or the T allele of the surface receptor for S1 of the angiotensin-converting enzyme 2 (ACE2) rs2285666 polymorphism exhibit a higher risk of severe COVID-19 [27]. In this Special Issue, Rodríguez Hermosa et al. found that subjects with alpha-1 antitrypsin deficiency (AATD) are at a higher risk of developing severe COVID-19 [28]. In fact, AATD levels below 116 mg/dL and the presence of a variant of the serine protein inhibitor-A1 (SERPINA1) gene, which could affect alpha-1 antitrypsin (A1AT) protein, were factors associated with the severe form of COVID-19 disease [28].

A study involving patients with chronic kidney disease, a vulnerable population, found that those with a lower estimated glomerular filtration rate and higher levels of Growth Differentiation Factor-15 (GDF-15) presented a higher risk of mortality associated with COVID-19 [29].

**Phenotyping** **of Post-COVID Pain**

Pain is an important but underestimated post-COVID symptom experienced by 15–20% of subjects after an acute SARS-CoV-2 infection [30,31]. In this Special Issue, a consensus paper on phenotyping post-COVID pain [32] proposed the application of the 2021 International Association for the Study of Pain (IASP) clinical criteria and grading system for classifying post-COVID pain symptomatology [33]. This consensus paper describes how post-COVID pain symptomatology can fulfill any of the phenotypes proposed by the IASP: nociceptive, neuropathic, nociplastic, and the mixed type [32]. In fact, based on current data, it seems that some patients suffering from post-COVID-19 condition will exhibit a pain phenotype with nociplastic characteristics. Nociplastic pain is defined by the IASP as “pain that arises from altered nociception despite no clear evidence of actual or threatened tissue damage causing the activation of peripheral nociceptors or evidence for disease or lesion of the somatosensory system causing the pain” [34]. Based on this definition, the presence of sensitization appears to be a primary mechanism associated with this phenotype. Several musculoskeletal chronic pain conditions are associated with pain sensitization [35]. Thus, nociplastic pain has also been associated with comorbid central-nervous-associated symptoms, e.g., poor sleep quality, fatigue, and cognitive–emotional disturbances, which are all present in post-COVID-19 condition.

**Others** 

In this last section, the remaining papers are summarized. Romanowska-Kocejko et al. observed that the dysregulation of metabolic processes in erythrocytes, in addition to endothelial and microvascular dysfunction, is associated with the decreased delivery of intracellular oxygen in patients with post-COVID-19 condition [36]. In accordance with the hypothesized endothelial problems, another study published in this Special Issue found that pulmonary embolism, as well as the use of a high-flow nasal cannula and prolonged hospitalization, is associated with reduced functional capacity and a higher likelihood of exertional desaturation in patients with post-COVID-19 condition [37]. Thus, the association of endothelial and microvascular dysfunction with reduced intracellular oxygen delivery may partly explain this post-COVID fatigue and limited functional capacity [36]. Similarly, the endothelial disfunction of the brain would explain the presence of post-COVID cognitive symptomatology [38]. In fact, the plethora of cardiovascular post-COVID symptoms that can be observed has been integrated in the term “vascular long-COVID” [39].

The last paper investigated differences in the immune response between patients with post-COVID-19 condition and those with interstitial pulmonary disease [40]. The study revealed a greater depletion of CD4 and natural killer cells in individuals with interstitial pulmonary disease, as well as an increase in CD8 cells. Furthermore, an increase in CD4 and CD8 cells, as a accentuating immune response, was observed in patients with post-COVID-19 condition [40].

As Guest Editor of this Special Issue, I would like to thank the reviewers for their comments, the authors for their valuable contributions, and the *Biomedicines* staff for their collective support and assistance during this process.
